# Health-related quality of life and family functioning of primary caregivers of children with down syndrome

**DOI:** 10.3389/fpsyt.2023.1267583

**Published:** 2023-12-14

**Authors:** Anna Rozensztrauch, Karolina Wieczorek, Iwona Twardak, Robert Śmigiel

**Affiliations:** Clinical Department of Pediatrics, Endocrinology, Diabetology and Metabolic Diseases, Wroclaw Medical University, Wroclaw, Poland

**Keywords:** intellectual disability, down syndrome, family, child, quality of life

## Abstract

**Background:**

Down Syndrome (DS; OMIM #190685), known as trisomy 21, is one of the most common genetic disorders in the human population and the commonest known cause of intellectual disability. The study was conducted to investigate the quality of life (QoL) of children with DS syndrome and its impact on family functioning.

**Purpose of study:**

To assess the quality of life of children with trisomy 21 and the impact of the disorder on the family.

**Methods:**

We used a cross-sectional questionnaire study. The respondents were 52 parents of children with trisomy 21. The following structured questionnaires were used: the PedsQL^™^ 4.0 Generic Core Scales, the PedsQL^™^ Family Impact Module and Study-Specific Questionnaire (SSQ).

**Results:**

The combined scores, with a mean value of approximately 55 out of a possible 100 points, indicated a significant impact of the child’s genetic defect on family functioning. In the overall QOL, the highest rated domain was physical functioning (x̅ =60.14; SD = 23.82) and the lowest was school functioning (x̅ =51.36; SD = 18.72). Better school functioning (*p* = 0.022) was reported for girls. The presence of reduced muscle tone also had a negative impact on the child’s functioning in the physical (*p* = 0.036), emotional (*p* = 0.011), psychosocial (*p* = 0.027) and overall QOL domains (*p* = 0.023).

**Conclusion:**

Overall, our results showed that the quality of life of children with trisomy 21 is impaired. There was a positive association between the child’s QOL and the QOL of their parents, as well as the general functioning of the child’s whole family. For this reason, an improvement in the QOL of parents and the family functioning is closely related to an increased QOL of the child. The continuous deepening of knowledge of QOL in individual trisomy 21 management allows for better preparation and ongoing care for the patients concerned.

## Introduction

Down syndrome (DS), known as trisomy 21, is one of the most common genetic disorders in the human population (1–3). Trisomy 21 is the commonest known medical cause of intellectual disability (4). Taking into account the availability of prenatal testing, birth statistics, and the number of pregnancy terminations performed, the frequency of this condition is estimated to be 1 in 600 to 1,200 live births worldwide (5). Trisomy 21 is a genetic disorder characterized by the presence of an extra chromosome or its part in the 21st pair of autosomal chromosomes (6). There are three mechanisms responsible for the occurrence of the syndrome: meiotic non-disjunction, errors in crossing over, and mitotic non-disjunction (7–9). Individuals with trisomy 21 are easily recognizable due to their characteristic appearance and the burden of various impairments that significantly affect their quality of life (QOL). Among the abnormalities, disorders affecting organs and even entire systems are distinguished. Pathologies most commonly involve the nervous system, circulatory system, including numerous heart defects, developmental and functional disorders of the digestive, respiratory, hormonal, urinary-reproductive, immune, and motor systems, as well as intellectual and psychomotor developmental disorders (10). A key problem concerning trisomy 21 is the presence of abnormalities in the central nervous system. Patients with trisomy 21 have been reported to have developmental abnormalities (3), neurodevelopmental alterations in the peripheral system, and also neurological and cognitive deficits (Table 1) (11–13). From early adulthood, patients with trisomy21 may present multiple conditions related to the progression of their ageing. The cardiovascular and immune systems seem to be the most affected, as is the brain, prompting some researchers to imply that trisomy 21 may be a segmental form of accelerated ageing (14). For a number of cases, these disabling diseases may include, for example, hearing and vision loss, episodes of epilepsy, depression and cognitive dementia. It is challenging to diagnose dementia in patients with trisomy 21, who may have cognitive alterations that are already occurring as a result of growth challenges (15).

**Table 1:** Age-related conditions in children and adults with trisomy 21 (11).

Comorbidity	Age
Infantile spasm	Infant
Learning, memory and speech problems	Child
Muscle hypotonia	Child
Multiple organ anomalies	Child
Congenital heart conditions	Child
Hearing impairment	Child/Adults
Thyroid disorders	Young/Adults
Sleep apnea	Young/Adults
Visual impairment	Adult
Epilepsy	Adult

As all children with trisomy 21 have a certain degree of intellectual disabilities (ID), they have been found to have a lower level of cognitive functioning than their peers (16). As a result of this, it is not surprising that this group also show lower levels of school functioning (17). In comparison to children with other ID, children with trisomy 21 demonstrate a greater degree of social competence (18). Nevertheless, children with trisomy 21 show increased problem behaviors and poorer social abilities (19). The presence of structural brain pathologies correlates closely with the ability to function in different life domains. Individuals with trisomy 21 exhibit attention deficit and divided attention deficit, are easily distracted, have difficulties in pursuing assigned tasks, and lack self-control and spontaneous activity. They also have significant problems with cognitive functions, such as abstract thinking, and comprehending and interpreting facts. Consequently, individuals with trisomy 21 have difficulty adapting to new situations and adjusting their behavior to the environment. Children with trisomy 21 have often mild to moderate mental impairment, although this is not a rule. Unfortunately, there is a downward trend in intelligence quotient with age (20).

The concept of quality of life (QOL) is the most commonly used term in sciences that focus on societal structure, psychological aspects, or even economic aspects of society. Due to the increasing interest in the term “quality of life” in medical sciences, the concept that is directly related to the health status of research participants was proposed – Health-Related Quality of Life (HRQoL). In 1993, WHO adopted a definition of quality of life as: “An individual’s perception of their position in life, in the context of the culture and value systems in which they live and in relation to their goals, expectations, standards, and concerns” (21). Studying the quality of life of patients in a specific medical field allows an assessment of the degree of functioning in various life domains in relation to limitations caused by the medical condition. QOL is directly affected by elements such as social functioning, physical and mental health, environmental and economic conditions. In the process of assessing quality of life, it is essential to consider each of these elements. Psychometric tests are among the tools that are used for assessing quality of life, enabling the creation of a patient’s health profile, as well as standardized questionnaires focusing on specific medical conditions. The selection of an appropriate questionnaire depends on the topic and purpose of the research conducted. QOL is not static and undergoes changes over time, hence the need for regular assessments of this phenomenon.

The existing research indicates that the QOL of children with trisomy 21 is lower than that of typically developing children (22) and, in particular, differences in levels of QOL occur in different areas. In general, children affected by trisomy 21 have a low physical well-being but a high emotional well-being (17, 23). Some studies indicate that QOL worsens with age (24), others report a higher QOL in young adults compared to adolescents (25), and some have found no age-related changes (26). Family resources, such as family income, were also associated with QOL in children with trisomy 21 (27).

The concept of family as a broadly understood structure or social institution encompassing a greater or lesser number of individuals enjoys considerable interest not only in social and human sciences but also in medical sciences. Family is associated with something natural, providing a sense of security and tranquility, thus creating an environment that enables the proper development of a child. It forms a kind of system of relationships among its members. Minczakiewicz et al. (28) describes the family as the pursuit of common goals, meeting the needs of each individual, and fulfilling the tasks entrusted to it by society. Having a family is associated with a burden of responsibility and obligation. The foundation of the bonds formed among family members includes a willingness to provide selfless help, a sense of responsibility towards each other, and the presence of emotions such as love, respect, and gratitude. Undoubtedly, health, the standard of living, and economic status are aspects that significantly influence relationships between close individuals. The functioning of a family proves to be particularly crucial when this harmonized structure faces a particular difficulty, such as illness or disability. With the birth of a child with trisomy 21, the struggle for their best possible life begins. Developmental, educational, and health challenges arise, a process of adapting to the new situation takes place, which can be accompanied by both positive and negative emotions. Often, this leads to a reorganization of the lives of all members of the family (29).

This study aims to assess the quality of life of children with trisomy 21 and the impact of the genetic defect on family functioning.

## Methods

### Setting

We performed a cross-sectional survey among parents of children with trisomy 21 between January 2022 and December 2022. These parents were included in the study if the following eligibility criteria were met: they are the biological parent of the child; their child has a diagnosis of trisomy 21 by a pediatrician and/or neurologist as clinically confirmed by molecular testing.

All of the participants provided written informed consent after receiving a thorough clarification of the procedures involved in the study.

The eligible parents were contacted and sent conventional paper questionnaires along with a self-addressed stamped envelope in which they were to return the filled-out questionnaire to the research team. The survey invitation package included a letter and an information pack explaining the study and the questionnaires to be completed. The anonymity of the participants was ensured by numbering each pack of questionnaires with a separate serial number rather than the name of the subject.

The study was conducted according to the guidelines of the Declaration of Helsinki and approved by the Institutional Review Board (or Ethics Committee) of Wroclaw Medical University (protocol code KB 35/2021 and 29 January 2021).

### Research instruments

The study was conducted using a Study-Specific Questionnaire (SSQ), and two standardized instruments, the Pediatric Impact Module PedsQL 2.0 questionnaire, which assesses the impact of a child’s health status on family functioning, and the Pediatric Quality of Life PedsQL 4.0 questionnaire, which assesses the overall quality of life of children, taking into account particular age groups of subjects (30–35).

### Study-specific questionnaire (SSQ)

The SSQ included the sociodemographic data of participants (e.g., age, sex, education, income) and disease-related data (comorbidities, presence of siblings, child’s age and sex).

#### PedsQL^™^-FIM

The PedsQL^™^-FIM assesses family functioning and is designed to measure the impact of chronic pediatric health conditions on the parents and family. The instrument consists of 36 items measuring parents’ self-reported functioning on six subscales: physical functioning (6 items), emotional functioning (5 items), social functioning (4 items), cognitive functioning (5 items), communication (3 items), and worry (5 items); two additional subscales measure parent-reported family functioning: daily activities (3 items) and family relationships (5 items). Each item is scored using a 5-point Likert scale from 0 (never a problem) to 4 (always a problem), which is then transformed into a 0-to-100 scale (0 = 100, 1 = 75, 2 = 50, 3 = 25, 4 = 0), with higher scores indicating better functioning.

#### PedsQL^™^ 4.0

The Pediatric Quality of Life Inventory (PedsQL) is a validated instrument for measuring quality of life (QOL) in children and adolescents aged 2–18 years with an acute or a chronic condition. The PedsQL 4.0 Generic Core Scales are self-report measures for children and proxy measures for their parents designed as a generic core measure for integration into disease-specific PedsQL modules. The instrument provides a questionnaire to evaluate four dimensions of functional outcome: physical functioning, emotional functioning, social functioning and school functioning. The directions contain a question about how much of a problem each of the items has been in the past month. A five-point response scale is used (0 = never a problem, 4 = almost always a problem). A higher score on the PedsQL instrument is an indication of a higher quality of life. The questionnaire has good psychometric properties (Cronbach’s alpha ranges from 0.66 to 0.93).

### Statistical analysis

The analysis of quantitative variables (i.e., expressed by number) was conducted by calculating the mean, standard deviation, median, quartiles, minimum and maximum values. The analysis of qualitative variables (i.e., not expressed by number) was conducted by calculating the number and percentage of occurrences of each value. Correlations between quantitative variables were analyzed using the Spearman’s correlation coefficient. The strength of the relationship was interpreted in accordance with the following scheme: |r| ≥ 0.9 – very strong relationship; 0.7 ≤ |r| < 0.9 – strong relationship; 0.5 ≤ |r| < 0.7 – moderately strong relationship; 0.3 ≤ |r| < 0.5 – weak relationship; |r| < 0.3 – very weak relationship (negligible). The quantitative variables were compared between two groups using the Mann–Whitney U test. A significance level of 0.05 was adopted in the analysis and all *p* values > 0.05 were interpreted as indicating significant relationships.

The statistical analysis was performed using the R software, version 4.1.2.

## Results

The parents of 53 children with trisomy 21 aged 2 to 18 years were involved in the study. The mean age of children was 6.48 (standard deviation SD = 4.56). There were 31 boys (58.49%) and 22 girls (41.51%). Based on the American Psychiatric Diagnostic and Statistical Manual of Mental Disorders (DSM), the largest group were children with mild intellectual disability and moderate intellectual disability in equal proportions (37.74%). There were 30 children (56.60%) with older siblings and 16 children (30.19%) were only children (Table 2). There were no statistically significant correlations between the guardian’s education level and the child’s functioning in any of the domains of QOL (all values: *p* > 0.05). The analysis revealed statistically significant correlations (*p* = 0.043) in domains such as emotional functioning and school functioning. There was significantly better functioning (*p* = 0.019) in the aforementioned domains in the group of children from families with a very good economic status.

**Table 2:** Characteristics of children with trisomy 21.

Feature		Mean (SD)	Min – Max
Child’s age		6.48 (SD = 4.56)	2–18
Feature		*n*	%
Child’s sex	Boys	31	58.49%
	Girls	22	41.51%
Child’s degree of intellectual disability	Profound	1	1.89%
	Severe	5	9.43%
	Moderate	20	37.74%
	Mild	20	37.74%
	Not specified	7	13.21%
Does (s)he have any siblings?	Is an only child	16	30.19%
	Has younger siblings	6	11.32%
	Has older siblings	30	56.60%
	Has older and younger siblings	1	1.89%

SD, standard deviation; Max, maximum value; Min, minimum value.

### QOL in children with DS

As shown by the data obtained, parents of children with trisomy 21 evaluated their overall quality of life at x̅ =57.51 (standard deviation, SD 17.56). The highest rated domain was physical functioning (PF) (PF; x̅ =60.14; SD = 23.82) and the lowest was school functioning (SCH) (SCH; x̅ =51.36; SD = 18.72) (Table 3). There was a statistically significant correlation (*p* = 0.022) between the child’s sex and their school functioning, and better functioning in this domain was observed in girls (x̅ =57.41; SD = 18.34) (Table 4).

**Table 3:** Average scores in the PedsQL^™^-4.0.

PedsQLTM-4.0	*N*	x̅	SD	Me	Min	Max	Q1	Q3
PF	53	60.14	23.82	65.62	0	100	50	71.88
EF	53	58.11	17.38	60	0	90	50	70
SF	53	56.7	22.27	55	0	100	45	70
SCH	43	51.36	18.72	50	0	90	42.5	65.83
PSF	53	56.02	16.37	55.77	0	85	48.08	63.46
Total score	53	57.51	17.56	59.72	0	86.96	47.62	67.86

x̅ mean; Me, median; Max, maximum value; Min, minimum value; *N*, number of respondents; Q1, first quartile; Q3, third quartile; SD, standard deviation; PF, physical functioning; EF, emotional functioning; SF, social functioning; SCH, school functioning; PSF, Psychosocial functioning.

**Table 4:** The child’s sex and quality of life in the PedsQL^™^-4.0.

PedsQL^™^-4.0		Child’s sex		*p*
		Boys (*N* = 31)	Girls (*N* = 22)	
Physical functioning	x̅ ±SD	55.11 ± 23.68	67.23 ± 22.67	*p* = 0.055
	Median	62.5	68.75	
	Quartiles	42.19–70.31	59.38–82.03	
Emotional functioning	x̅ ±SD	58.55 ± 14.44	57.5 ± 21.2	*p* = 0.993
	Median	60	60	
	Quartiles	50–67.5	46.25–73.75	
Social functioning	x̅ ±SD	54.19 ± 19.37	60.23 ± 25.89	*p* = 0.261
	Median	55	62.5	
	Quartiles	42.5–65	45–83.75	
School functioning	x̅ ±SD	47 ± 18.1	57.41 ± 18.34	*p* = 0.022 *
	Median	45	58.33	
	Quartiles	35–55	50–69.17	
Psychosocial functioning	x̅ ±SD	54.21 ± 13.83	58.58 ± 19.46	*p* = 0.173
	Median	55	60.77	
	Quartiles	46.67–60	48.75–74.04	
Total score	x̅ ±SD	54.43 ± 15.66	61.84 ± 19.47	*p* = 0.055
	Median	55.43	61.96	
	Quartiles	46.74–61.93	51.49–73.41	

x̅, mean; SD, standard deviation, *p – statistical significance.

There was no statistically significant correlation between the guardian’s education level and the child’s functioning in any of the PedsQL^™^ 4.0 generic core dimensions (all values: *p* > 0.05).

The study revealed statistically significant correlations (*p* = 0.043) in domains such as emotional functioning and school functioning. There was significantly (*p* = 0.019) improved functioning in the aforementioned domains in the group of children from families with a very good economic status.

There were several comorbidities in the study sample. Due to the format of the multiple-choice questions posed to the respondents, the impact of each answer had to be analyzed separately. Therefore, the analysis focused on diseases that were present in at least five of the children surveyed (Table 5).

**Table 5:** Assessment of parents’ functioning in various domains.

PedsQL– family impact	*N*	x̅	SD	Median	Min	Max	Q1	Q3
PF	53	53.38	19.98	50	0	91.67	41.67	66.67
EF	53	54.15	20.8	50	0	95	40	65
SF	53	54.72	23.54	56.25	0	100	37.5	68.75
CF	53	64.53	24.42	65	0	100	50	80
C	53	56.92	25.98	50	0	100	41.67	75
W	53	41.98	20.95	40	0	90	25	55
DA	53	44.97	23.87	50	0	100	33.33	58.33
FR	53	62.03	24.49	65	0	100	50	80
FF	53	55.64	21.87	56.25	0	90.62	46.88	71.88
Total score	53	54.4	18.79	53.47	0	86.11	42.36	67.36

x̅, mean; Me, median; Max, maximum value; Min, minimum value; *N*, number of respondents; Q1, first quartile; Q3, third quartile; SD, standard deviation; PF, physical functioning; EF, emotional functioning; SF, social functioning; CF, Cognitive functioning; C, Communication; W, Worry; DA, Daily activities; FR, Family relationships; FF, Family functioning.

There was no statistically significant correlation between PedsQL^™^ 4.0 generic core dimensions and the incidence of immunodeficiencies (x̅ =59.1; SD = 16.05), hearing and vision impairments (x̅ =61.98; SD = 15.31), or congenital heart defects. However, statistically significant correlations were found between the overall QOL and the presence of thyroid dysfunction (*p* = 0,036; x̅ =61,17; SD = 21.61). Children without this condition exhibited better overall QOL. The presence of reduced muscle tone also negatively affected the functioning of the child in the physical (PF, *p* = 0.036; x̅ =72.81; SD = 20.32), emotional (EF, *p* = 0.011, x̅ =69.55; SD = 11.5) and psychosocial domains (PSF, *p* = 0.027, x̅ =64.77; SD = 11.59), and overall QOL (*p* = 0.023, x̅ =67.83; SD = 14.03). The results also indicate better physical functioning (PF, *p* = 0.013, x̅ =65.38; SD = 21.07) in children without defects in the digestive tract.

### The family impact

The Family Impact questionnaire provided data, the analysis of which made it possible to assess the functioning of parents of children with trisomy 21 in individual domains and the functioning of the family as a whole (Table 6). The combined results, with a mean value of around 55 out of a possible 100 points, indicated a significant impact of the child’s genetic defect on how the family functioned. The analysis of the results revealed that the best functioning of the respondents was related to the cognitive domain (CF; x̅ =64.53; SD = 24.42) and family relationships (FR; x̅ =62.03; SD = 24.49). The worst functioning was observed in domains such as worry (W; x̅ =41.98; SD = 20.95) and daily activities (DA; x̅ =44.97; SD = 23.87).

**Table 6:** Summary of the prevalence of comorbidities in study sample.

Comorbidities	*n*	%*
Leukemia	1	1.89%
Epilepsy	3	5.66%
Pulmonary hypertension	1	1.89%
Immunodeficiencies	16	30.19%
Hypothyroidism	28	52.83%
Reduced muscle tension	42	79.25%
Iron absorption problems	1	1.89%
Hypertrophied tonsils	1	1.89%
Different leg lengths	1	1.89%
Persistent inflammation of the sinuses	1	1.89%
Clubfeet	1	1.89%
Hip joint defect	1	1.89%
Defects in the digestive tract	15	28.30%
Hearing impairment, visual impairment	36	67.92%
Genitourinary tract defects	4	7.55%
Joint laxity	1	1.89%
Cryptorchidism	2	3.77%
Congenital heart defects	31	58.49%
West syndrome	1	1.89%
Significant delay in speech development	1	1.89%
Absence of associated defects	3	5.66%

*N*, number of respondents.

The analysis did not show a statistically significant correlation between the child’s age and their QOL/their functioning in individual domains (all *p* > 0.05, QOL FIM total score *r* = 0.227; *p* = 0.103), (PF; *r* = 0.199; *p* = 0.154), (EF, *r* = 0.142; *p* = 0.311), (SF, *r* = 0.21; *p* = 0.131), (SCH *r* = 0.187; *p* = 0.23), (PSF, *r* = 0.221, *p* = 0.112).

The analysis of the data revealed that the QOL of children is significantly and positively correlated with the QOL of their parent and family functioning in each domain (*p* < 0.05). This means that the better the QOL of a child with DS, the better the QOL of their parents and the overall functioning of the family. Therefore, an increase in the parental QOL and family functioning is connected with an increase in the child’s QOL (Table 7).

**Table 7:** Analysis of the correlation between the child’s quality of life and family functioning.

	FIM: physical functioning	FIM: emotional functioning	FIM: social functioning	FIM: cognitive functioning	FIM: communication	FIM: worry	FIM: daily Activities	FIM: family Relationships	FIM: overall Parental Quality of Life	FIM: family Functioning	FIM: total score
PedsQL: PF	*r* = 0.437, *p* = 0.001 *	*r* = 0.572, *p* < 0.001 *	*r* = 0.527, *p* < 0.001 *	*r* = 0.298, *p* = 0.03 *	*r* = 0.508, *p* < 0.001 *	*r* = 0.37, *p* = 0.006 *	*r* = 0.513, *p* < 0.001 *	*r* = 0.524, *p* < 0.001 *	*r* = 0.512, *p* < 0.001 *	*r* = 0.609, *p* < 0.001 *	*r* = 0.57, *p* < 0.001 *
PedsQL: EF	*r* = 0.563, *p* < 0.001 *	*r* = 0.578, *p* < 0.001 *	*r* = 0.56, *p* < 0.001 *	*r* = 0.445, *p* = 0.001 *	*r* = 0.445, *p* = 0.001 *	*r* = 0.445, *p* = 0.001 *	*r* = 0.471, *p* < 0.001 *	*r* = 0.556, *p* < 0.001 *	*r* = 0.602, *p* < 0.001 *	*r* = 0.605, *p* < 0.001 *	*r* = 0.603, *p* < 0.001 *
PedsQL: SF	*r* = 0.427, *p* = 0.001 *	*r* = 0.603, *p* < 0.001 *	*r* = 0.502, *p* < 0.001 *	*r* = 0.323, *p* = 0.018 *	*r* = 0.523, *p* < 0.001 *	*r* = 0.451, *p* = 0.001 *	*r* = 0.49, *p* < 0.001 *	*r* = 0.458, *p* = 0.001 *	*r* = 0.527, *p* < 0.001 *	*r* = 0.532, *p* < 0.001 *	*r* = 0.56, *p* < 0.001 *
PedsQL: SCH	*r* = 0.639, *p* < 0.001 *	*r* = 0.547, *p* < 0.001 *	*r* = 0.622, *p* < 0.001 *	*r* = 0.551, *p* < 0.001 *	*r* = 0.499, *p* = 0.001 *	*r* = 0.365, *p* = 0.016 *	*r* = 0.47, *p* = 0.001 *	*r* = 0.538, *p* < 0.001 *	*r* = 0.617, *p* < 0.001 *	*r* = 0.555, *p* < 0.001 *	*r* = 0.644, *p* < 0.001 *
PedsQL: PF	*r* = 0.631, *p* < 0.001 *	*r* = 0.735, *p* < 0.001 *	*r* = 0.638, *p* < 0.001 *	*r* = 0.48, *p* < 0.001 *	*r* = 0.638, *p* < 0.001 *	*r* = 0.443, *p* = 0.001 *	*r* = 0.569, *p* < 0.001 *	*r* = 0.658, *p* < 0.001 *	*r* = 0.699, *p* < 0.001 *	*r* = 0.72, *p* < 0.001 *	*r* = 0.729, *p* < 0.001 *
PedsQL: total score	*r* = 0.602, *p* < 0.001 *	*r* = 0.741, *p* < 0.001 *	*r* = 0.68, *p* < 0.001 *	*r* = 0.438, *p* = 0.001 *	*r* = 0.647, *p* < 0.001 *	*r* = 0.448, *p* = 0.001 *	*r* = 0.623, *p* < 0.001 *	*r* = 0.616, *p* < 0.001 *	*r* = 0.686, *p* < 0.001 *	*r* = 0.715, *p* < 0.001 *	*r* = 0.725, *p* < 0.001 *

*r* – Spearman’s correlation coefficient, * statistically significant relationship (*p* < 0.05).

## Discussion

This study indicates that trisomy 21 not only impacts children, but also influences the whole family functioning, reducing the QOL and considerably influencing the realization of social roles and reducing the overall QOL. Taking care of a child with trisomy 21 often lasts for many years, permanently altering the traditional model of family functioning. In the United States, an increase was observed in life expectancy for individuals with trisomy 21 from 26 years in 1950 to 53–58 years in 2010 (7). Similar studies were conducted in England and Wales, where the average life expectancy in 2011 was 51 years (36).

The study sample indicates that the overall QOL of children with trisomy 21, as assessed by their parents, was exactly 57.51 out of a possible 100 points.

According to the research conducted by Rojnueangnit et al. (17) and Xanthopoulos et al. (37), the QOL of children with trisomy 21 is rated lower compared to typically developing children. The overall average scores of the general QOL range from 65 to 70 points (out of 100). Researchers also noted the diversity of results in specific domains, where emotional functioning is considered the highest-rated domain, with scores that do not differ significantly from those of typically developing children. A slightly different observation was made by Shields et al. (23), according to whom the school functioning domain is the best-rated aspect. As for the lowest-rated domain, attention should be given to school functioning (17), physical functioning (23), and social functioning (23, 37). Moreover, the reduced level of QOL in the physical well-being domains can be interpreted in terms of the greater risk of having a number of medical comorbidities, such as cardiac and respiratory complications (17, 24, 37). The results of our own research indicate that the highest-rated domains in children with trisomy 21 were physical functioning and emotional functioning, while the lowest-rated domain was school/pre-school functioning, which slightly deviates from the results described above. However, they still refer to the same aspects of life, which undoubtedly draws attention to existing deficiencies in these domains.

No significant correlation between the age of the child and their overall quality of life was revealed by the analysis of the research data. Similar results were obtained by Katsian et al. (38), where the correlation analysis of the variables did not reveal statistically significant relationships with any of the domains or with the summary scale assessing overall QOL. On the other hand, Shields et al. (23) found that teenagers have a lower quality of life compared to younger children, especially in the aspects of social life, peer interactions, and even physical well-being. According to the research by Lee et al. (24), the average scores in the domain of emotional functioning and social functioning were significantly higher in the group of children aged 4–5 years compared to the average scores in the groups of children aged 13–21 years and 6–12 years. There are some reports implying that despite many successes in interacting with others, individuals with trisomy 21 may experience certain difficulties in this area as they age. This could be related to expectations that individuals with trisomy 21 may not be able to meet. Researchers also mention that intellectual disability or behavioral problems could be the reasons for disturbances in functioning in these domains.

The research material demonstrated a relationship between school functioning and the child’s sex. The results were significantly better for girls. However, this relationship does not find confirmation in other studies (39, 40). Further extensive research in this area is considered necessary.

No significant relationships were found when analyzing the relationship between the QOL of a child with trisomy 21 and having siblings. This result differs from the findings by Hodapp et al. (41) in somewhat older studies. They emphasize the presence of better relationships between siblings in families with a child with trisomy 21 compared to families with siblings that are raised facing other disabilities. These families also demonstrate better organization, greater care, and support for all family members. Pasqualucci et al. (42) confirm these results and also draw attention to the lower occurrence of jealousy among siblings towards parental attention.

The analysis also focused on the impact of parents’ education level on the overall quality of life of a child with trisomy 21. The findings did not show any statistically significant relationships between the variables studied.

In this study, there was a statistically significant relationship between the family’s economic status and the child’s functioning in the emotional domain and school functioning domain. Children’s functioning in these areas was better when the family status was classified as “very good.” Having a child with a disability involves additional expenses related to diagnostic and therapeutic procedures (36). It often happens that one of the parents resigns from their professional activity to take care of the child, which can certainly impact the family’s later economic conditions. The results of our own research indicate that in the vast majority of cases, the family’s economic status was classified as “sufficient” or “good.” Only 5 respondents classified it as “very good.” According to the research report from 2017–2018 (25), in 99.2% of the families surveyed, all expenses in the aforementioned scope are covered from their own resources. The high costs associated with raising a child with trisomy 21 often force parents to seek additional sources of finance. Unfortunately, this can lead to the loosening of family ties and, consequently, may also affect the child’s level of functioning.

Respondents most commonly reported low muscle tone, hearing and vision impairments, and heart defects as the most prevalent conditions coexisting with trisomy 21. The coexistence of these conditions worsens the overall QOL of children, as well as their functioning in the physical, emotional, and psychosocial domains. These results confirm research conducted in Thailand by Rojnueangnit et al. (17), which aimed to assess the quality of life of children with trisomy 21 and the quality of healthcare provided to them.

The study also analyzed the overall functioning of the family and, consequently, the parental QOL in the context of their child’s genetic defect. The families of the children with trisomy 21 surveyed cope best in domains such as cognitive functioning and family relationships. However, the biggest challenges for them are worries and daily activities. Moreover, it was noted that parents of a child with trisomy 21 are well-adjusted in the social functioning aspect and can rely on significant support from others, which they often use in stressful situations. Similarly, the study by De Faria et al. (43) revealed that parents of children with DS rated their QOL as “good” and were satisfied with it.

The study conducted allows for the conclusion that trisomy 21 as a combination of various disorders and conditions significantly affects the QOL and functioning of children and their families. This assessment is the result of the analysis of our own research and other authors’ studies, which have shown that both parental quality of life and family functioning undergo a significant deterioration.

In this study, there was a positive relationship between the child’s QOL and the QOL of their parents, as well as the overall functioning of the child’s entire family. This means that when the QOL of one party improves, the other responds in the same way.

The continuous development of knowledge about the QOL in individual medical units allows better preparation and improvement of the ongoing care of the patients concerned. Furthermore, it is possible to provide appropriate living conditions and functioning for individual patients and their families. Genetic defects are and will continue to be a topic that affects the lives of some of us. It is essential to provide support for these individuals in various aspects of life.

The findings of this study need to be considered in terms of its limitations. The first limitation is that due to the specificity of the disease, the QOL was only assessed from the parents’ perspective and that we excluded other sources of data, for instance, self-reports or teachers’ reports. The parents’ self-reports may not fully represent their children’s experience. Due to the prevalence of this condition, the occurrence, causes and treatment of trisomy 21 are eagerly addressed by many researchers. However, there is a lack of studies relating to the assessment of the QOL of the patients themselves, especially children. Most research in this area usually centers on assessing parents’ perspectives. In this case, it seems essential to measure and assess the QOL of the affected individuals themselves, which would allow their needs and expectations to be identified. The other limitation is that QOL was evaluated on the basis of generic scales; a child-specific and disease-specific questionnaire should be used in future studies. Another possible limitation of the study was the small sample size. Therefore, the results should be interpreted with certain caution. Nonetheless, part of these results are of great importance and definitely prompt further research and the setting up of multi-center collaborations with associations of family members with children with trisomy 21.

## Conclusion

Overall, our results showed that the quality of life of children with trisomy 21 is impaired. There was a positive association between the child’s QOL and the QOL of their parents, as well as the general functioning of the child’s whole family. For this reason, an improvement in the QOL of parents and the family functioning is closely related to an increased QOL of the child. The continuous deepening of knowledge of QOL in individual trisomy 21 management allows for better preparation and ongoing care for the patients concerned.

## Data availability statement

The original contributions presented in the study are included in the article/supplementary material, further inquiries can be directed to the corresponding author.

## Ethics statement

The studies involving humans were approved by the study was conducted according to the guidelines of the Declaration of Helsinki and approved by the Institutional Review Board (or Ethics Committee) of Wroclaw Medical University (protocol code KB 35/2021 and 29 January 2021). The studies were conducted in accordance with the local legislation and institutional requirements. Written informed consent for participation in this study was provided by the participants' legal guardians/next of kin.

## Author contributions

AR: Conceptualization, Formal analysis, Investigation, Methodology, Project administration, Resources, Supervision, Validation, Visualization, Writing – original draft, Writing – review & editing. KW: Data curation, Formal analysis, Investigation, Project administration, Resources, Software, Writing – original draft. IT: Project administration, Writing – original draft. RŚ: Supervision, Visualization, Writing – review & editing.
